# The relationship between neural phase entrainment and statistical word-learning: A scoping review

**DOI:** 10.3758/s13423-023-02425-9

**Published:** 2023-12-07

**Authors:** Guro S. Sjuls, Nora N. Harvei, Mila D. Vulchanova

**Affiliations:** Department of Language and Literature, Norwegian University of Science and Technology, Dragvoll alle 6, 7049 Trondheim, Norway

**Keywords:** Statistical learning, Word-learning, Neural entrainment, Neural tracking, Frequency-tagging

## Abstract

Statistical language-learning, the capacity to extract regularities from a continuous speech stream, arguably involves the ability to segment the stream before the discrete constituents can be stored in memory. According to recent accounts, the segmentation process is reflected in the alignment of neural activity to the statistical structure embedded in the input. However, the degree to which it can predict the subsequent leaning outcome is currently unclear. As this is a relatively new avenue of research on statistical learning, a scoping review approach was adopted to identify and explore the current body of evidence on the use of neural phase entrainment as a measure of online neural statistical language-learning and its relation to the learning outcome, as well as the design characteristics of these studies. All included studies (11) observed entrainment to the underlying statistical pattern with exposure to the structured speech stream. A significant association between entrainment and learning outcome was observed in six of the studies. We discuss these findings in light of what neural entrainment in statistical word-learning experiments might represent, and speculate that it might reflect a general auditory processing mechanism, rather than segmentation of the speech stream per se. Lastly, as we find the current selection of studies to provide inconclusive evidence for neural entrainment’s role in statistical learning, future research avenues are proposed.

**Supplementary Information:**

The online version contains supplementary material available at 10.3758/s13423-023-02425-9.

## Introduction

Statistical learning (SL), typically defined as the ability to extract regularities and patterns from the environment (Saffran et al., 1996b), is assumed to be a central mechanism in the process of acquiring knowledge for humans and other species (for a review, see, e.g., Schiavo & Froemke, 2019). SL has been observed in several domains, one of which is language development (Saffran & Kirkham, 2018). Given that natural speech is a continuous stream of sounds with no clear markers of the onset and offset of each word (Lehiste, 1960), identifying word boundaries poses a challenge for infants and second-language learners alike. SL is argued to identify regularities in the underlying structure of the input, and thus provide the cognitive system with important linguistic cues, which aid the language acquisition process (Siegelman & Frost, 2015). As first demonstrated by Saffran and colleagues (1996a), who investigated infants’ sensitivity to word boundaries, SL can facilitate the segmentation of the speech stream. In turn, this process enables retention and storage of the word form, to which word meaning can be associated (Saffran, 2003).

In speech, some syllables have a higher transitional probability (TP), meaning that they have a higher probability of occurring together (e.g., if they are part of the same word). Syllables in adjacent words, on the other hand, have a low TP between them. Through SL, the word unit can be segmented from the speech stream, as TP is higher for syllables within words than between words (Saffran et al., 1996a, b). For example, the TP is higher for the syllables within each of the words in *happy pony* (e.g., between *ha* and *py*, and *po* and *ny*) than for the syllables between each word (between *py* and *po*), as *happy* could be followed by any other word. In other words, sensitivity to these regularities allows for smaller perceptual units, such as syllables, to be grouped into larger perceptual units, such as words, which can be stored in memory. In their original study, Saffran and colleagues (1996a) found that infants utilized TP in extracting information about syllable statistics, a finding supported by numerous later studies of statistical word-learning, across populations of language learners (e.g., Isbilen & Christiansen, 2022; Kuhl, 2004; Maye et al., 2002; Saffran et al., 1996b; Saffran & Kirkham, 2018).

**Fig. 1:**
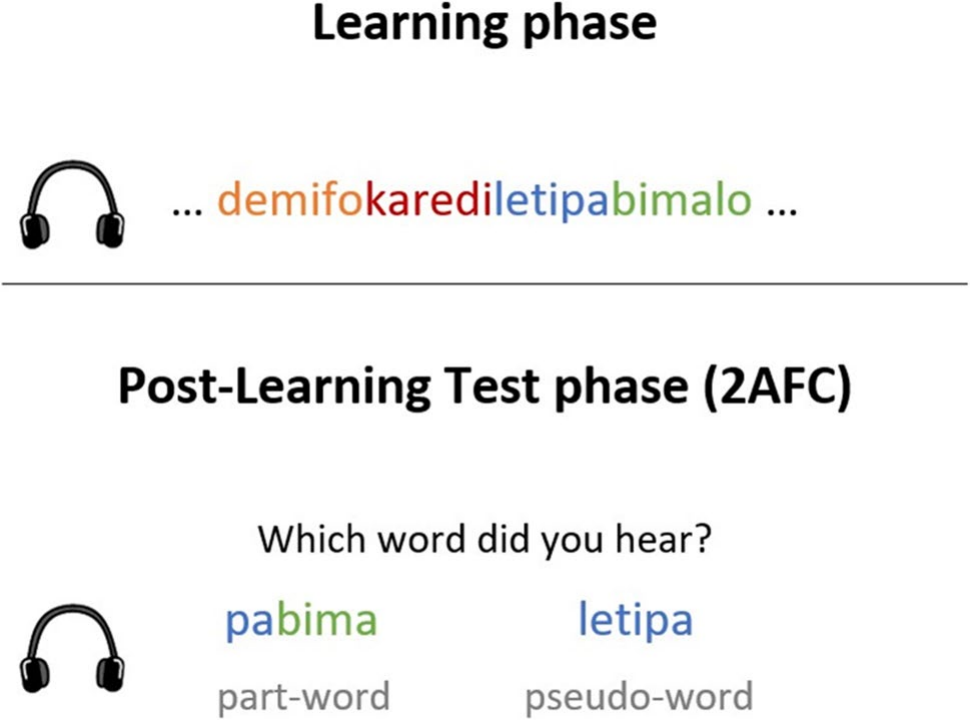
Example of statistical word-learning experiment. The learning phase and 2AFC explicit postlearning test of statistical word-learning. Participants are presented with a stream of syllables with pseudowords cued by a statistical regularity (e.g., high TP between adjacent syllables within one pseudoword). Then, participants are presented with the pseudoword and a foil (part-word) consisting of syllables across the pseudoword boundaries. (Color figure online)

The majority of SL experiments utilize humans’ sensitivity to statistical regularities, such as TPs, and follow the same general study design. In the language domain, these studies typically consist of a learning phase, where participants are exposed to a continuous speech stream consisting of repeated trisyllabic pseudowords. These pseudowords usually do not contain any clear acoustic cues to word boundaries, such that the only cue to word segmentation is the statistical regularity between the syllables (e.g., high TP between all syllables within a pseudoword or between the first and last syllable of a pseudoword^1^). The degree to which participants successfully segment out the larger units is then measured with a test following the learning phase (Fig. 1). Several postlearning tests have been developed to assess the learning outcome. In explicit tests, participants must actively recall or recognize the unit of segmentation. For adults, a two-alternative forced-choice (2AFC) task is often used (e.g., Lopez-Barroso et al., 2011; Saffran et al., 1997; Saffran et al., 1996b; Turk-Browne et al., 2005). More recently, and following concerns for tapping individual differences, statistically induced chunking recall (SICR) tasks have been employed, where participants are asked to explicitly repeat the experimental units (e.g., Isbilen et al., 2020; Kidd et al., 2020). Moreover, participants’ familiarity with the unit of segmentation can also be indirectly assessed (for instance, through measures of reaction time; e.g., Hunt & Aslin, 2001). For infants, the duration of visual fixation to test items is often measured (e.g., Shi & Werker, 2001). In later years, studies have begun utilizing neuroscientific techniques such as event-related potentials (ERPs) and functional magnetic resonance imaging (fMRI) to study the neural mechanisms underlying SL and to assess familiarity to test items (e.g., Cunillera et al., 2009; Karuza et al., 2013; McNealy et al., 2006).

One limitation of the traditional SL paradigms is that the learning outcome typically is assessed *after* the learning phase, and as such, it cannot inform on the time course of learning (Batterink & Paller, 2017). Batterink and Paller (2017) suggest that the SL process has two components: (1) perceptual binding of the individual units, such as syllables, into larger perceptual units, such as words (the word-identification component), and (2) storing these units for later use (the memory-storage component). Further, they argue that poor performance on the postlearning test might reflect individual differences in the word-identification component *or* it might reflect individual differences in the memory-storage component (such as long- or short-term memory capacity). While the memory-storage component is necessary for retaining information, the word-identification component is suggested to be more revealing about the statistical segmentation process itself, as it reflects the perceptual binding of syllables into words. Yet, with traditional postlearning tests, the relative contribution of each of the components cannot be teased apart. However, there is a theoretical possibility that individual differences in the word-identification component contribute to individual variability on postlearning tests (Batterink & Paller, 2017). To address this issue, Batterink and Paller (2017) developed a paradigm to specifically test the word-identification component, by analyzing the intertrial phase coherence (ITPC) of electroencephalogram (EEG) signals recorded during the learning phase.

The firing neurons of the brain elicit electrical potentials which can be measured with high temporal resolution by means of noninvasive EEG and magnetoencephalogram (MEG), and invasive electrocorticography (ECoG) techniques. The obtained complex time series can be analyzed in multiple ways, such as fast changes related to experimentally introduces events (event-related potentials [ERPs]), or over larger timescales. Neural activity oscillates at specific frequencies, as clusters of cortical neurons enter cycles of exhibitory and inhibitory states (Goswami, 2019), and the dynamics of the oscillations can be assessed with frequency or time frequency approaches, over both longer and shorter timescales. These approaches can be used to inform on the synchronization or *entrainment* of the neural signals to properties of the input signal (van Bree et al., 2021). Entrainment is, in essence, estimated by extracting epochs from the continuous neural signal, which is time locked to the onset of an aspect of the input signal of interest, and phase coherence (or clustering) of the phase angles across epochs is then computed. The phase coherence, typically measured from 0 (not phase locked) to 1 (fully phase locked), at specific frequencies of interest can then be extracted (Kabdebon et al., 2015).^2^

Although studies of rhythmic neural activity go far back (Adrian, 1944), it has received increasing interest over the past decade. Moreover, recent studies consistently show a tendency for neural oscillations to entrain to ongoing rhythmic stimuli. One example of such neural entrainment is to the quasirhythmic features of the temporal waveform of speech (Ding et al., 2016), from rapid fluctuations at the phonemic level, to slower fluctuations at the prosodic phrase level. Alignment of neural activity in the corresponding delta (0.5–3.5 Hz), theta (4–8 Hz) and low-gamma (>35 Hz) frequency bands could underlie the processing of prosodic phrases, syllables, and (sub)phonemic units of speech (Giraud & Poeppel, 2012). In other words, different linguistic units are simultaneously tracked by the brain, which could facilitate more sufficient processing and prediction of upcoming events in the environment.

**Fig. 2:**
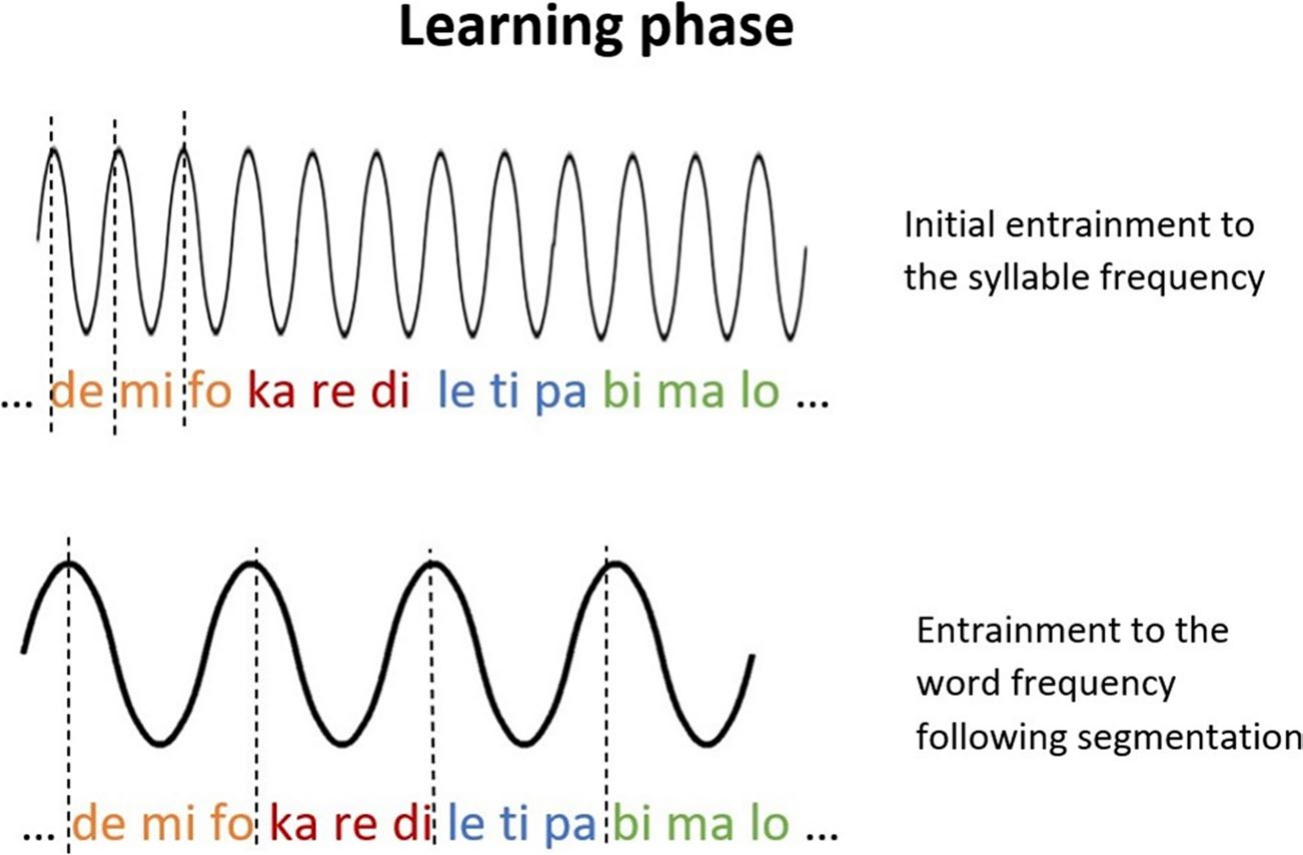
Measuring the word-identification component. Perceptual shift with exposure to structured input, as reflected in shift in neural entrainment. Illustration adapted from Batterink and Paller (2017), with permission

As the neural response corresponds to the frequency of basic perceptual (linguistic) units, it could be sensitive to SL. According to Batterink and Paller’s (2017) account, a perceptual shift from syllables to words could be detected as an initial neural entrainment to the syllable units, which would then shift towards the word units with exposure to the structured input (Fig. 2). Neural entrainment could thus tap into the underlying mechanism for extracting statistical information (the word-identification component). In comparison, when employing traditional postlearning measures of SL, the relative contribution of the word-identification component and the memory-storage component is unknown.

Batterink and Paller (2017) estimated the relative entrainment to the word and syllable frequency with exposure to the structured speech stream, compared with the entrainment to a stream of random syllables. Further, the relationship between entrainment and the scores on postlearning tests was explored to estimate the degree of observable differences in the word-identification component, and whether this was reflected in the postlearning outcome. They argue that if there were no differences in entrainment between individuals, it would imply similar sensory processing of regularities across individuals. In this case, observed individual differences on the postlearning tests could be attributed to the memory-storage component. If, however, there were individual differences in the word-identification component, as reflected in neural entrainment to words over syllables, then this should, at least partially, be reflected in the observed individual differences on the postlearning test. The results of their study will be described in more detail in the Results-section.

Similar approaches have also been previously tested (in, e.g., Buiatti et al., 2009; Kabdebon et al., 2015),^3^ and several others have since adopted the same approach as a measure of online SL. Here, we aim to address studies of neural entrainment to the underlying pattern during the learning phase of language-related SL experiments and its relationship to the learning outcome. In other words, whether differences in the word-identification component—namely, the online processing of regularities—are reflected in the learning outcome. This is a relatively new area of research on SL, and as such, we aim to scope the literature for relevant research and comment on similar and different aspects of these studies, in terms of their study design. Moreover, scoping the existing literature will give insight into potential gaps in the research field.

Following these aims, the overarching review question is whether and to what extent neural phase entrainment is a useful online measure of statistical word-learning. The review question is addressed by the following review objectives: (1) identify and assess the body of evidence on the use of phase entrainment as an online measure of auditory linguistic SL; (2) identify study-specific characteristics related to study population, study design, analyses of neural data, the results of the entrainment analyses and evidence of the association between neural entrainment and learning outcome; (3) synthesize the results; (4) identify gaps in the literature. We found a scoping review to be the most appropriate choice as the literature on this topic has not yet been reviewed. By adopting a scoping review approach, we stayed open to the possibility of discovering data extraction points of interest while conducting the review and subsequently to address these emerging topics of interest.

## Methods

### Protocol and registration

The review protocol was first uploaded to the Open Science Framework (see Data and Materials) on January 27, 2022. Amendments to the original protocol, including the description and rationale for updates, was uploaded to the same project on June 11, 2022, along with the same protocol as a clean document without markings and comments. A preliminary search was conducted, where no current or underway reviews on the topic were identified.

### Eligibility criteria

Studies that included and reported results from human individuals of any age and background were considered. We considered experimental paradigms where participants were familiarized with a natural or artificial language in the auditory domain, followed by a test of their learning of a given probabilistic rule or pattern embedded in the language. In addition, studies had to include an analysis of the phase entrainment of the neural activity to the stimuli presented during the learning phase. Purely power-based frequency-tagging approaches were not included in the present review (e.g., Buiatti et al., 2009; Ramos-Escobar et al., 2021). This decision was made as the current scoping review aims at providing an initial insight into this relatively new subfield of SL, while follow-up studies are encouraged to elaborate on this. Including such studies in future reviews will provide results that are highly complementary to those presented here.

After receiving comments during the peer-review process, adjustments to the eligibility criteria were made, to only include studies that specifically assessed the statistical relationship between entrainment and the learning outcome. In our original sample of papers, some studies tested the learning outcome and entrainment, and assumed an association between the two if both were significant. However, such results do not necessarily speak for a relationship between the two variables. Thus, we only included papers that statistically tested, and explicitly reported on, the association between these variables. The eligibility criteria were thus redefined to better allow for addressing the research questions, a strategy consistent with the advantages of the scoping review methodology.

### Information sources and search strategy

The sources of information were limited to original empirical studies published in peer-reviewed journals with English as the language of publication. There were no restrictions on the search with regard to the timeframe of the studies. Initially, a search was conducted in PsychINFO, to assess search terms contained in the title, abstract and keywords of relevant articles. After deciding on appropriate search terms, a full search strategy was developed. Information was sought in the three relevant electronic databases PsychINFO, PubMed, and Web of Science, on January 31, 2022.

### Selection of articles

**Fig. 3:**
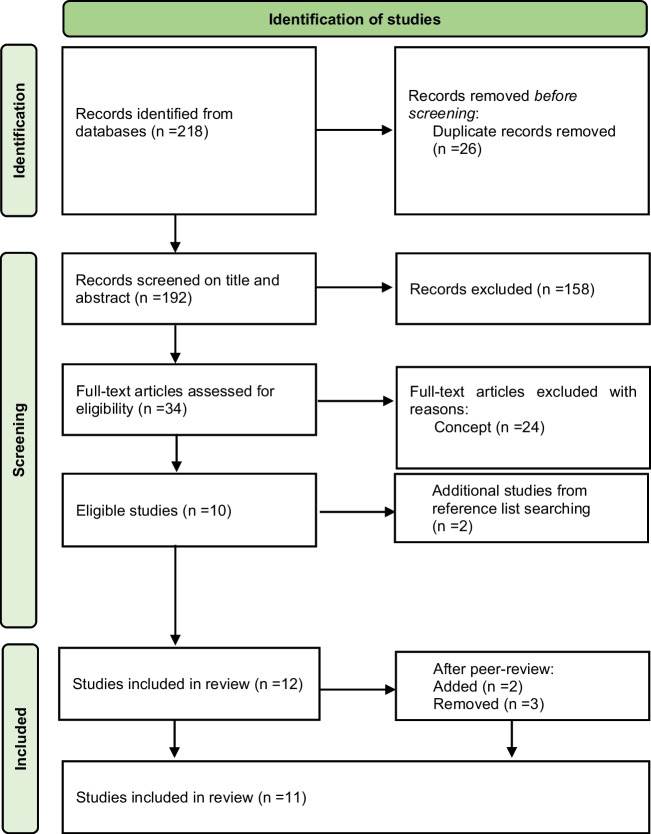
Study selection flow chart. The study selection process, reported in a flow diagram, as proposed by the PRISMA-ScR protocol, in-line with the JBI (Tricco et al., 2018). The database search yielded a total of 218 articles, from which 26 were removed as duplicates. From the 192 articles that were screened on title and abstract, 158 were excluded as they did not meet one or more eligibility criteria. The interrater reliability agreement for this section of the screening was substantial (Cohen’s kappa = 0.63). Disagreements among the reviewers were due to Reviewer 2 being slightly stricter as compared with Reviewer 1, who tended to include more articles. After thorough discussion, 34 articles were agreed upon for screening on full text. Of these articles, 24 were excluded. The interrater reliability agreement for this section of the screening was substantial (Cohen’s kappa = 0.65). Disagreements among the reviewers were due to Reviewer 1 being slightly more inclined to exclude articles as compared with Reviewer 2. After thorough discussion, 10 articles were selected to be included in the study. The citations of the final selection of articles were screened, and two additional articles were included (Kabdebon et al., 2015; Pinto et al., 2022) (Cohen’s kappa = 1). At every step of the screening process, the interrater reliability agreement was substantial, namely above the predetermined level of Cohen’s kappa = 0.61. A total of 12 records were initially included. After revising the eligibility criteria and carefully reading the included papers, after peer review, three papers were removed (Batterink & Zhang, 2022; Elmer et al., 2021; Getz et al., 2018) and two were included (Henin et al., 2021; Moreau et al., 2022)

Following the search, all identified citations (title, authors, abstract) were uploaded and managed in EndNote software (Version 21; EndNote, 2013). Two reviewers independently screened titles and abstracts to assess eligibility for inclusion in the review, after duplicates were removed. Both reviewers then screened the full text of the papers included. The reviewers’ decisions at each stage of the selection process were compared, aiming for a substantial inter-rater reliability (Cohen’s kappa ≥ 0.61). Any disagreements were discussed and are reported. After the selection process, the reference lists of the eligible studies were searched for additional relevant articles (Fig. 3).

Reasons for excluding articles at the different stages were primarily related to the eligibility criteria not being met with regards to the concept. For example, articles which did not report on original empirical findings; did not study SL; did not use phase entrainment as a neural measure or as a measure of SL; did not include a postlearning measure of SL; did not study language, but, for example, SL in the visual domain or of musical sequences; did not assess the relationship between entrainment and learning outcome (see Eligibility Criteria). Some articles were close to being included in the final selection of articles. For instance, Batterink and Choi (2021) met most inclusion criteria but did not report on original empirical data, as they performed reanalyses of data reported in previous studies. Generally, their results and conclusions remain unaltered from those originally reported. Therefore, only data from the original articles were included in the current review.

Initially, 12 articles were included. As previously mentioned, the criteria were adjusted after receiving peer review on the original manuscript, to only include papers in which the association between entrainment and the learning outcome had been statically addressed. After careful reading of all included papers, three were removed for not sufficiently meeting the criteria. However, one new, recently published paper was added, in addition to one paper suggested by the reviewers that did meet the inclusion criteria. Thus, a total of 11 papers were included in the scoping review (see Fig. 3 for details).

### Data items and extraction process

Data were extracted from the articles in accordance with the review objectives, following a data extraction template (Supplementary Fig. 1). Various points were revised, following information we found to be of interest while conducting the search, during the scoping of the articles and during the peer-review process. Some assumptions were made regarding the data extraction: If not otherwise specified, the studies were assumed to study a typical population; only the number of participants who underwent both the familiarization phase and postlearning test was extracted; if not otherwise specified, no pauses were assumed between the words of the learning phase. The data were independently extracted by one reviewer.

## Results

Overall, the studies follow the same general design. Neural activity is recorded during a learning phase, where participants are presented with a stream of structured linguistic input containing a statistical regularity cuing segmentation. The neural response during the structured learning phase is often compared with a control condition that does not contain such regularities. Then, whether the stream was correctly segmented is assessed through administering an implicit or explicit postlearning test of SL. Some aspect of the neural analysis is then related to the participants’ learning outcome (see Table 2).

In several of the studies, the Word Learning Index (WLI) is computed to quantify individual sensitivity to the underlying pattern (Batterink & Paller, 2017). The WLI is calculated by dividing the neural entrainment at the frequency of the segmentation unit (e.g., word frequency) by the frequency of the units which make up the segmentation unit (e.g., syllable frequency). If participants become sensitive to the underlying pattern, the WLI should (1) be higher in the experimental condition containing statistical cues to segmentation, compared with a control condition that does not contain such cues, as indexed by a relatively greater entrainment at the word frequency and a weaker entrainment at the syllable frequency for the experimental, compared with the control condition, or (2) increase over time, as indexed by a greater WLI as a function of time (e.g., as compared between blocks of exposure).

Here, data were extracted from each article to identify study-specific characteristics (see Tables 1 and 2), in accordance with the data extraction template (Supplementary Fig. 1).

**Table 1:** Study samples and neurophysiological method

Record	Mean age/ Age range^1^	Population type^2^	Sample size ^3^	Recording of neural activity	Number of recording electrodes	Control Condition/ Comparison to Assess Neural Entrainment
Batterink & Paller, 2017	21 years	Typical	23	EEG	64	Random syllable stream
Batterink & Paller, 2019	22 years	Typical	19; 25	EEG	64	-
Batterink, 2020	21 years	Typical	21	EEG	64	Surrogate data (null distribution of entrainment values)
Choi et al., 2020	175 days	Typical (full-term)	18	EEG	64	-
Henin et al., 2021	35 years	Epilepsy patients	17	ECoG	3689	Random syllable stream
Kabdebon et al., 2015	8 months	Full term; premature 8 months after birth; premature 8 months after term	42	EEG	64	Surrogate data (null distribution of entrainment values)
Moreau et al., 2022	10 years; 20 years	Typical	56; 24	EEG	32	Surrogate data (null distribution of entrainment values)
Ordin et al., 2020	18-26 years	Typical	34	EEG	27	Resting-state recordings
Pinto et al., 2022	25 years	Typical	40	EEG	64	Acoustically controlled random syllable stream
Smalle et al., 2022	24; 20 years	Typical	36 (18; 18); 60 (20; 20; 20)	EEG	27	Random syllable stream
Zhang et al., 2021	24; 21 years	Dyslexic; typical readers	18; 18	EEG	64	Random syllable stream

^1^Age range is reported in table if mean age is not reported in the article. Age is summed to nearest integer

^2^If otherwise not specifically specified, the included records were assumed to have studied typical populations

^3^Number of participants for which both neural and behavioral data was available for analyses. Kabdebon et al. (2015) split the participants in three groups (full term infants, premature 8 months after birth, and premature 8 months after term), but it is not specified how many infants from each group are represented in the analyses concerning both the learning and test parts, other than it being 42 infants overall. In Smalle et al. (2021), two experiments are reported, in which there were 18 participants in each group (control vs dorsolateral prefrontal cortex TMS) in experiment 1, and 20 participants in each attention group of experiment 2 (high-load, low-load and no-load). In Pinto et al. (2022), data for 27 of the 40 participants was available for the target-detection task

Abbreviations/ symbols: EEG = electroencephalogram; ECoG =electrocorticography; “;” = per condition/experiment/group, if applicable

**Table 2:** Study design and outcome

Record	Statistical regularity^1^	Segmentation unit^2^	Number of seg. units	Syllable duration	Learning phase duration ^3^	Pauses between seg**.** units^4^	Post-learning tests^5^	Entrainment measure associated with post-learning test	Post-learning tests for which evidence of association to entrainment was observed ^6^
Batterink & Paller, 2017	High TP within words	Trisyllabic pseudo-words	4	300 ms	12 minutes (3)	-	Rating task; Target-detection task	WLI	RT on target-detection task
Batterink & Paller, 2019	High TP within words	Trisyllabic pseudo-words	4	260 – 300 ms	12 minutes (3)	-	Rating task; Target-detection task	WLI	RT on target-detection task
Batterink, 2020	High TP within words	Trisyllabic pseudo-words	6	Mean = 235	21 minutes (3)	-	2AFC (item-level recognition; overall recognition)	Word frequency entrainment to word-items; Average word frequency entrainment at the individual-level	2AFC (item-level entrainment and item-level recognition; average entrainment at the individual-level and overall recognition)
Choi et al., 2020	High TP within words	Trisyllabic pseudo-words	4	300 ms	2 – 6.6 minutes (1)	-	Preferential looking-time paradigm	WLI	Novelty preference for nonwords
Henin et al., 2021	High TP within words	Trisyllabic pseudo-words	4	250ms	~2 minutes (5)	-	2AFC	Word frequency entrainment	-
Kabdebon et al., 2015	Non-adjacent dependencies	Trisyllabic pseudo-words	9 (across three families of words)	232 ms	~2 minutes (1)	25 ms between words	PLV (difference in entrainment to the part and rule-words)	Syllable and word frequency entrainment	PLV (for the syllable frequency)
Moreau et al., 2022	High TP within words	Trisyllabic pseudo-words	4	300 ms	6 minutes	-	Rating task; 2AFC; Target-detection task	WLI	-
Ordin et al., 2020	High TP within words	Trisyllabic pseudo-words	12	240 ms	18 minutes (1)	-	2AFC	Word frequency entrainment (the first part subtracted by the last part of the learning phase)	-
Pinto et al., 2022	High TP within words	Trisyllabic pseudo-words	6	250 ms	3.22 minutes (3)	-	2AFC; Target-detection task	Average word frequency (and its harmonics) entrainment; Group comparison based on whether or not individual word frequency (and its harmonics) entrainment was observed	RT on target-detection task (for group comparison)
Smalle et al., 2022	High TP within words	Trisyllabic pseudo-words	4	250 ms	5 minutes (4)	-	2AFC	WLI	-
Zhang et al., 2021	High TP within words	Trisyllabic pseudo-words; natural Dutch words	4; 4	300 ms	2.5 minutes (6; 2)	-	2AFC	Maximum entrainment at the word frequency	-

^1^The type of statistical regularity from which the participants could learn. The stimuli in Kabdebon et al. (2015) consisted of a AXB structure of non-adjacent dependencies. In Batterink (2020) eleven syllables were combined to create six pseudo-words. As some syllables occurred in more than one pseudo-word, the TP within words varied somewhat, similarly to in natural languages

^2^Stimuli presented during the learning phase

^3^The total learning phase duration, over number of blocks (in parentheses), per experimental condition. In Smalle et al. (2021), the experimental condition (structured stream) was presented for 20 minutes, while the control condition (random syllables) was presented for ~5 minutes

^4^ Note that the duration of the pause in Kabdebon et al. (2015) is subtle and not consciously perceived by adults (Peña et al., 2002)

^5^ Post-learning test(s) for which the association to measures of neural entrainment was/were assessed

^6^ The post-learning tests that were significantly correlated with entrainment during learning. Line indicates that no significant relationship was observed. Note that in Batterink (2020), average entrainment at the participant-level did significantly predict the 2AFC results, but only if item-level entrainment was not included in the model. When participantlevel entrainment was the only predictor in the model, it became significant but performed more poorly than item-level entrainment as predictor

Abbreviations: TP = transitional probability; 2AFC = two-alternative forced choice; RT = reaction time; ERP = event-related potential; WLI = Word Learning Index; PLV = phaselocking value; seg. = segmentation

### Summary of studies

#### Online statistical learning

Batterink and Paller (2017) aimed at characterizing the word-identification component of statistical learning, as described in the introduction, as well as its time course, the degree of variability among individuals and its relation to the learning outcome, as measured by performance on postlearning tests. While EEG was recorded, participants were exposed to two syllable streams, where the experimental stream contained repeated pseudowords and the control stream contained nonrepeated random syllables, before their performance on the postlearning tests was assessed. There were peaks in entrainment at the two frequencies of interest (word and syllable frequencies). The entrainment scores were used to calculate the WLI, to assess the relative entrainment to the word frequency for the experimental versus the control condition, and over blocks of exposure. WLI significantly differed between the conditions over all blocks, with greater scores in the experimental condition, which significantly increased across expose blocks. The change in WLI is reflected in a significant interaction between WLI and frequency, with greater entrainment at the word frequency and less entrainment to the syllable in the experimental condition compared with the control. For the experimental condition, reaction time on a target-detection task (an indirect performance-based measure) was significantly associated with the WLI, while performance on an explicit rating task was not significantly correlated. Interestingly, however, the WLI for the control condition was also significantly correlated with the learning outcome on the target-detection task, and the WLI for the experimental and control conditions were significantly correlated. Furthermore, they observed an increase in the WLI for the structured stream, a learning curve, when assessing each exposure block, while a similar finding was not obtained for the control condition.

Following the same line of work, Batterink (2020) studied whether greater neural entrainment to the word frequency is more robustly associated with successfully learned pseudowords, compared with pseudowords that were poorly learned. This study was carried out to address whether neural tracking of the linguistic structures embedded in the speech stream reflects the learning of word items, or rather an individual trait. Such an individual trait could, for instance, be a more general, enhanced sensitivity to temporal patterns, which could be a potential predictor of SL abilities, which furthermore could be what is reflected in entrainment to the word frequency. Based on the EEG signal obtained during the learning phase, peaks in entrainment at the word and syllable frequencies were observed. Tested against a surrogate, null distribution of phase-locking values, which represented entrainment to randomly selected syllable triplets, entrainment to the word items during the learning phase was significantly different (for different word types). The different word types were differently tracked, as entrainment significantly varied between them. Furthermore, participants performed above chance on the postlearning task, and both entrainment to word items and variability in the pseudoword duration positively and significantly predicted the recognition at the item level on the postlearning task. When including entrainment at the item level and average entrainment at the participant level in a prediction model, for frequencies corresponding to pseudowords, only entrainment at the item-level significantly predicted recognition of the specific words. When the average entrainment at the participant-level at the word frequency (not item-level entrainment) was included as predictor, the model significantly predicted item-level recognition, but it performed more poorly than the model including entrainment at the item level. Overarchingly, average overall entrainment to the word frequency predicted the overall recognition scores on the postlearning test. In addition, entrainment at the item level, overall words, increased with exposure during the first block of exposure, where the words with the highest recognition score also showed significant increases, while the words with the lowest recognition scores showed a decrease. Taken together, it is suggested that entrainment to individual words reflects the degree of recognition of these words, rather than other intraindividual aspects of processing which may also predict SL performance.

Pinto et al. (2022) aimed to test the robustness of the association between neural entrainment to words and explicit and implicit postlearning measures. Furthermore, whether the predictions can be made at the group and individual level was of interest, as typically used measures of SL usually have low sensitivity at both, and in particular at the individual level. The within-subject correlation between explicit and implicit measures is also often low. Contrary to other studies in the current review, Pinto and colleagues controlled for acoustic features that might have impacted the relationship between postlearning measures and entrainment to the stream. Namely, the control and experimental stimulus might contain acoustic confounds at the frequency of the segmentation unit (e.g., the word). In these cases, the statistical regularity is neither the sole cue to segmenting the stream nor for neural entrainment at the word frequency to occur. Here, a position-controlled stimulus was used as a control, to give the control and experimental stimuli similar modulation spectrums, as effects at the word frequency could not be eliminated completely from the experimental stimuli. Thus, a control with similar acoustic properties would allow for estimating the extent to which the acoustic confounds contribute to the neural entrainment. With regard to the explicit 2AFC task administered after exposure to the streams, accuracy levels were not above chance at the group level, and very few participants performed above chance at the individual level. For the implicit target-detection task, reaction times and accuracy were significantly faster and higher for pseudowords compared with nonwords at the group level. However, at the individual level, 70% had significant effects for either accuracy or RT, but only very few showed significant effects on both. The explicit and implicit task scores did not show a within-subject correlation. With regards to the entrainment, peaks at the syllable and word frequencies, as well as at the 1^st^ and 3^rd^ harmonics^4^ of the word, were observed for both conditions at the group level. When comparing the structured and control streams, significantly larger entrainment was found to the word frequency for the structured stream (anecdotally supported by Bayes factor) and at the 3^rd^ harmonic of the word (strongly supported by Bayes factor), compared with the control. Entrainment to the syllable frequency was significantly lower for the structured stream, compared with the control. When assessing changes across three exposure blocks, all effects of condition were found to be present already in the first block (corresponding to ~3.2 minutes). At the individual level, entrainment at the word frequency and its harmonics were included as frequencies of interest, as the effect of SL could manifest at either. At these frequencies, significant effects of condition were observed, with larger effects in the structured compared with the control stream in 31% of the cases. However, the effect was spread between the different frequencies of interest. The effect of lower entrainment at the syllable frequency, which was observed at the group level, was significant only for a small portion of participants at the individual level. The relationship between these findings and the postlearning measures was assessed by correlating the average entrainment at the three frequencies of interest with each postlearning measure, and by separating the participants into two groups based on whether significant entrainment was observed (as described above). The first analysis (at the individual level) yielded no significant results, while the second found the group that entrained to have faster reaction times on the target detection task, although there were no differences on the hit rate of the same test or the accuracy on the 2AFC.

Henin et al. (2021) were interested in the neural substrates which support SL and explored the role of cortical and hippocampal brain regions during the acquisition of statistical regularities, as these regions have been shown to be influenced by SL post learning. In their investigation of auditory linguistic SL, participants’ responses when presented with a structured stream of pseudowords and a random stream of syllables were measured by means of intracranial electrocorticography (ECoG) recordings, in epilepsy patients. During exposure, an online SL task was conducted. After exposure, participants were asked to perform a recognition test (2AFC) of the pseudowords compared with part words. The participants did not perform significantly above chance on the test (although a separate cohort of healthy subjects did). Significant entrainment was observed for certain electrodes at the syllable frequency for both the structured and the random stream, and at the word frequency in the structured stream. This response emerged rapidly, and 16/17 patients exhibited a significant entrainment effect. To further examine the effects and their localization during exposure to the structured stream, electrodes showing significant entrainment to both the word and syllable frequencies were compared with electrodes showing a significant effect at the word frequency only. The results indicated that electrodes showing a significant effect at both word and syllable frequencies were primarily located in the superior temporal gyrus, with smaller clusters in motor cortex and pars opercularis, while the word-only effects were located in the inferior frontal gyrus and the anterior temporal lobe. The authors propose that this reflects the anatomy of the auditory processing hierarchy, suggesting that word-only effects might arise from higher-order stages of processing. In terms of the association to the learning outcome, no significant correlation was observed between the maximum entrainment at the word frequency in the structured stream and the postlearning test. A significant correlation was, however, observed between the online SL task (performed during learning) and the maximum entrainment at the word frequency.

Ordin et al. (2020) were interested in the type of regularities which trigger SL in the context of the ongoing debate on the role of TPs in the segmentation of continuous input. They studied whether adults extract the statistical information by attending to the high TP between frequently co-occurring syllables within words, which is clustered together into units (clustering mechanisms), or by attending to the low TP marking the edges between words and extract the words as whole units (boundary-finding mechanisms). Furthermore, the learning outcome—namely, what is endorsed as a legitimate constituent during the postlearning test—was investigated. During the learning phase, participants were presented with a stream of pseudowords which allowed for the emergence of *phantoms* as a perceptual unit. Namely, if both ABC and UBZ is presented in the stream, ABZ could be recognized during the postlearning test although it was never presented during the learning phase. For the test phase, a 2AFC test, with recognition of pseudowords, phantoms, and nonwords (here: random syllable sequences) was included, with the rationale that clustering mechanisms would lead to higher endorsements of phantoms, because frequently co-occurring syllables are learned. Moreover, boundary-finding mechanisms would lead to better recognition of pseudowords (as syllables between boundaries are extracted as whole constituents). Following the same line of thinking, entrainment to the word frequency should gradually arise if a boundary-finding mechanism is at play, while entrainment at the boundaries between frequently co-occurring syllable pairs, leading to an increase at the frequency of the syllable with exposure to the stream, should arise if clustering mechanisms are at play. Behaviorally, participants preferred pseudowords over phantoms and nonwords, and phantoms over nonwords, more often than expected by chance. With regards to the entrainment, two significant peaks (at the syllable and word rate) were detected, compared with a resting-state recording. At the word frequency, a gradual increase occurred, while entrainment at the syllable frequency remained the same. To assess the relationship between the learning phase and the learning outcome, the entrainment estimates during the first part of exposure (assumed to reflect initial exposure) was subtracted from the last part of exposure (assumed to reflect word-level knowledge), and correlated to the learning outcome (i.e., when pseudowords, phantoms and nonwords were recognized). None of the correlations turned out significant. The results are interpreted to suggest that low TPs between words are used to insert boundaries between words in the continuous speech signal, rather than being in support of a chunking mechanism.

#### Developmental trajectory

Choi et al. (2020) studied neural entrainment to pseudowords to investigate online SL and later ability to discriminate previously encountered pseudowords from nonwords, in preverbal infants. They observed peaks in entrainment at the word frequency and syllable frequency, and a significant increase in WLI as a function of time, an effect which was most pronounced over approximately the first 90 seconds of the learning phase. In addition, infants looking times were significantly longer for nonwords, reflecting a novelty preference, and their proportional preference score was positively and significantly correlated with the final WLI value. It is proposed that infants with higher WLI scores might be more efficient learners and as such more likely to display increased focus towards the novel nonwords compared with familiar structures. Lastly, the trajectory of the WLI was compared with previously obtained results with adults (from Batterink & Paller, 2017), and no significant difference between the learning trajectories was observed.

In another study on infants, Kabdebon et al. (2015) explored infants’ ability to compute long-distance dependencies, to estimate whether there exists a gap between the age at which infants can compute the high TP of adjacent syllables in pseudowords (at 8 months) and the time at which they can do nonadjacent statistical computations. Infants were exposed to a stream of trisyllabic pseudowords consisting of nonadjacent dependencies following AXB structure, and the entrainment during learning to a surrogate dataset was estimated. Subsequently, infants’ ability to recognize the pattern was assessed as their neural response (phase-locking value [PLV]) to rule-words (following the pattern) and part words (not following the pattern). These results were related to the entrainment to the syllabic and word frequencies during learning. During learning, significant peaks in entrainment to the syllabic and word frequencies compared with the surrogate data were observed, and there was no significant difference between groups (preterm, full term). For the entrainment during the test phase, there were no significant difference between rule words and part words at the word frequency, but higher entrainment for part words than rule words at the syllable frequency was found. When assessing the correlation between these results and the entrainment during learning, no significant results were obtained for the word frequency, but a significant negative correlation was observed for the syllable frequency. In other words, a stronger syllabic entrainment during learning was correlated with a larger difference between conditions during the test phase. However, no correlation was observed between entrainment to the word frequency during learning and the learning outcome.

Moreau et al. (2022) aimed to compare SL between adults and children. Given previous findings and limitations in the literature, they investigated the possibility that children have an advantage over adults. As such, neural entrainment, implicit and explicit postlearning tests were employed in the study. With regards to the behavioral results, children and adults similarly rated pseudowords presented in the structured stream as most familiar, followed by part words and nonwords. On the 2AFC task, both groups performed above chance, but not significantly different from each other. Both children and adults showed progressively shorter reaction times on the target detection task, and adults were significantly faster than children. The reaction time priming effect was significantly larger for adults (relative to the baseline reaction time, the relative change in speed to predictable syllables was larger). In terms of the neural entrainment, significant peaks at the word and syllable frequencies were observable for both groups, compared with surrogate data in which no entrainment occurred. Their analysis was further focused on the time course of learning for each group, in which adults displayed a significant increase in the WLI over time, driven by a significant increase at the word frequency and a significant decrease at the syllable frequency. For children, only the word-frequency peak showed a significant change, namely an increase over time. When assessing the surrogate (control) data, entrainment to the word frequency started at a similar level, but increased significantly less over time. However, neural entrainment as measured with the WLI did not significantly correlate with any of the postlearning measures of SL, for either group.

#### Cognitive functions and atypical populations

Batterink and Paller (2019) investigated the role of attention in SL and its influence on the word-identification and memory-storage components. Specifically, they aimed at answering several related questions, such as whether learners can become sensitive to the underlying structure of a stream of pseudowords in the absence of attention towards the stream, whether focused attention facilitates the ability to extract the underlying pseudowords, and whether attention towards the stream affects differentially the word-identification component (as measured with EEG) and the memory storage component (as measured postlearning tasks). Participants were randomly assigned to one of two groups, in which they were either instructed to have full attention to the experimental stimuli during the learning phase, or to complete a demanding visual task during the learning phase. Both groups showed significant learning effects on a familiarity rating task, but there was no significant difference between the two groups. Reaction time on a target detection task was, however, significantly faster for the full attention group. With regard to the neural entrainment, peaks at both syllable and word frequencies were observed, and the entrainment was significantly greater at the syllable frequency for the full attention group. No significant differences were found with regard to the time course of the word frequency entrainment between the two groups. Based on these results, it is suggested that a reduction in attention toward the speech stream does not seriously impact learners’ ability to utilize the underlying statistical cues to segment out word forms, as measured with the EEG approach. Furthermore, the WLI across the learning phase significantly predicted the reaction time on the target detection task.

Smalle et al. (2022) tested the hypothesis that mature cognitive mechanisms can constrain implicit SL mechanisms, by depleting cognitive control mechanisms in adult participants by means of inhibitory theta-burst stimulation, a form of transcranial magnetic stimulation (TMS; Experiment 1) and a dual working-memory task (Experiment 2). It was predicted that cognitive depletion would increase the participants’ implicit, auditory word-segmentation abilities, as higher-level cognitive functions associated with declarative memory (explicit learning) have been found to interfere with implicit learning mechanisms. That is, if higher-level memory functions interfere with implicit learning, it could be an explanation of why infants and children acquire language with less effort than adults, for whom these functions are more developed. The study assessed this in two different experiments, in which participants were exposed to a stream of repeating pseudowords during which EEG was recorded, before being tested on their recognition of the embedded pseudowords. In the first experiment, where TMS-induced disruption of the dorsolateral prefrontal cortex was used, participants in both the control and experimental groups performed above chance on the postlearning task, and the disrupted group performed significantly better on accuracy. In the second experiment, where participants first completed a cognitive load task with either high or low load (or no load, for the control group), all groups performed above chance. The high and low load groups performed significantly better than the control group. With regards to the entrainment to the underlying structure, compared with a random syllable control stream, significantly increased entrainment to the word and decreased entrainment to the syllable rates were found in both experiments. In Experiment 1, the TMS significantly increased the WLI. However, cognitive load in Experiment 2 did not affect the WLI. They did not find a significant correlation between the WLI and the results on the postlearning test (for all groups across experiments, nor for the control groups). However, as they did find enhanced implicit learning when inducing cognitive depletion, the authors argue that the lack of correlation might be due to the neural and behavioral measures assessing two different mechanisms of SL—namely, the perceptual binding, as measured with EEG, and the memory component, as measured with the postlearning tasks. Thus, they suggest that enhanced word recognition under cognitive depletion can be ascribed to the memory-storage component, but not the word-identification component.

Zhang et al. (2021) assessed the time course of SL in a group of dyslexic readers as compared with a group of typical readers, to provide a dynamic neural perspective on the difficulties with extracting statistical structures as earlier reported for dyslexic readers. In addition to a stream of pseudowords, entrainment to real words was assessed to examine whether the neural representation of the pseudowords embedded in the stream resemble that of familiar words. The relationship between entrainment to pseudoword and real word structures, and behavioral measures of SL and skills associated with reading ability (phonological awareness and [non]-symbolic visual-verbal conversion skills) were explored. With regards to the neural measures, the entrainment analysis showed that a peak at the syllable frequency was comparable across the conditions (random stream of syllables, structured stream and real-word stream), while only the structured pseudoword stream and real-word stream displayed a peak at the word frequency. The tracking of syllables was also comparable across the groups and blocks of exposure, namely, it developed in a similar way over time for typical and dyslexic readers alike. The neural tracking of pseudowords, on the other hand, took significantly less time of exposure to reach its maximum for typical, compared with dyslexic, readers. For typical readers, the structured pseudoword stream showed a significantly higher word-rate entrainment than the random stream, an effect which developed rapidly and dropped off in the two last expose blocks. For the dyslexic group, on the other hand, a significant effect of condition, but not exposure block, was observed. In other words, there was no significant increase in entrainment to the word frequency (to the structured stream) over time, although there was a significant difference between the structured and random conditions overall. However, post hoc analysis of the effect of condition showed that a significant difference in entrainment to the word frequency between the structured and random stream was evident only in the two last blocks of exposure. To evaluate the degree of entrainment to the pseudowords for each group, the maximum entrainment to real-word structures was used as a benchmark which was compared with individually determined maximum entrainment to the word frequency in the structured stream. There was no significant difference between the groups in terms of their maximum entrainment to real words, and no significant difference between the entrainment to the word frequency in the real-word versus structured stream condition, although there was a significant difference between the groups in terms of entrainment to the structured stream, with the dyslexic group displaying significantly less entrainment. On the postlearning task, typical readers performed significantly above chance (on accuracy in the recognition task), while dyslexic readers did not. There was also no difference between the groups on the average reaction time or recognition accuracy of the test. In terms of the association between the maximum entrainment at the word frequency in the real word and structured stream condition, and the learning outcomes, no significant correlation was observed for either type of entrainment. To summarize, the time course of entrainment to the pseudowords in the structured stream developed slower for dyslexic readers, and maximum entrainment to these structures did not significantly correlate with learning outcome. Furthermore, the most direct test of the study hypothesis (Condition × Block × Group interaction)—namely, that tracking of pseudowords develops less efficiently in the dyslexic group, did not reach significance.

### Similarities and differences across studies

#### Study design

In terms of the study design, these studies are fairly similar, with most articles reporting on a healthy adult population, by analyzing data from 18 to 60 participants (per group; Table 1). Two articles report on data from infants (Choi et al., 2020; Kabdebon et al., 2015), one reports on children (and adults; Moreau et al., 2022), and three articles report on atypical populations (Henin et al., 2021; Kabdebon et al., 2015; Zhang et al., 2021). Most studies used EEG (most often with 64 electrodes) to record neural activity (Table 1).

With regards to the study and stimuli design, most of the studies used the traditional design of trisyllabic pseudowords (number = 4–12; mean syllable duration = 267 ms) presented continuously during the learning phase (duration = ~2–21 minutes, divided over blocks or not). Note however that natural words from the participants’ native language were included in Zhang et al. (2021; Table 2). All studies used high within-word TP as a cue for segmentation during the learning phase, except for in Kabdebon et al. (2015), where they looked at non-adjacent dependencies. This was also the only study with pauses included between segmentation units (Table 2).

For the postlearning test phase, all studies used behavioral measures to test the learning outcome, except for Kabdebon et al. (2015), where entrainment to target and nontarget words were used. Target detection was the most frequently used test of implicit SL, while 2AFC was the most frequent test of explicit SL (Table 2). In a target-detection task, participants are expected to (speedily) detect syllables occurring in the pseudowords that are embedded in the structured syllable stream presented during the learning phase (Batterink & Paller, 2017). Reaction times (RTs) are measured to assess implicit statistical knowledge (Batterink et al., 2015; Moreau et al., 2022). With regard to the type of tests used, two studies included only implicit tests, five included only explicit tests, and five included both. Taken together, implicit tests were thus included in seven cases, while explicit tests were included in 10 cases (Table 2).^5^

#### Neural entrainment to the embedded structure

Typically, entrainment to the embedded structure was investigated by (1) assessing whether entrainment to the word frequency or the WLI was greater in the experimental condition compared with a control condition, or (2) assessing whether entrainment to the word frequency or the WLI increased over time (in some cases compared with a control condition). This affects the degree to which the analyses in the different studies can statistically inform on whether significant entrainment at the frequency of interest is present, regardless of the relationship with the postlearning measure. However, descriptively, all the studies appear to find some entrainment to the hidden structure.

The control conditions consisted of either a random syllable stream, to which entrainment during the structured stream could be compared, or a null-distribution of entrainment values, to which the entrainment values to the structured stream could be compared. In the first case, the participants were presented with the random stream in a similar manner to the experimental condition(s)—namely, during the experimental procedure. Here, syllables were concatenated in a pseudorandom fashion, without any distinct higher-order structure, with the only requirement being that syllables do not repeat consecutively. This ensured a lack of structured patterns in the syllable sequence. In the second case, however, the control entailed creating entrainment values by reshuffling the EEG data to create a surrogate data set (representing the null hypothesis that entrainment values are not higher for nonwords than for randomly selected syllable triplets) to which the real data was compared. If the observed entrainment values exceeded a given value (e.g., the 95^th^ percentile of the surrogate distribution, it was considered an indication of significantly greater entrainment to the word frequency compared with random syllables; see Table 1).

All studies using WLI as an index of learning found peaks at the syllable and word frequencies (Batterink, 2020; Batterink & Paller, 2017, 2019; Choi et al., 2020; Zhang et al., 2021), although they are not always statistically assessed. A significantly higher WLI in the experimental compared with the control condition is however also reported in some studies (Batterink & Paller, 2017; Smalle et al. 2022). A WLI increase for the experimental condition is also observed (Batterink & Paller, 2017; Moreau et al., 2022 [for adults]; Zhang et al., 2021), although not always compared with a clear control condition (Batterink & Paller, 2019; Choi et al., 2020).

Furthermore, TMS induced cognitive depletion significantly increased the WLI, but not when cognitive depletion was induced by cognitive load (Smalle et al., 2022). Here, significantly increased entrainment at the word frequency and a decline in entrainment at the syllable frequency was found in both experiments (TMS- and cognitive-load-induced cognitive depletion). Moreau et al. (2022) observed a significant increase in WLI over time for adults, driven by an increase at the word frequency and decrease at the syllable frequency. Children, on the other hand, did not show a significant decrease at the syllable frequency, which might explain why the subsequent WLI did not significantly change over time. Still, entrainment at the word frequency significantly increased in children, similarly to adults.

In terms of the studies that did not employ the WLI, significant peaks at the syllable and word frequencies were observed in the experimental condition (Kabdebon et al., 2015; Ordin et al., 2020). Similarly to the trends in the WLI studies, Ordin et al. (2020) found entrainment at the word, but not at the syllable, frequency, to significantly increase over time, while Kabdebon et al. (2015) found entrainment at both frequencies to significantly increase. Pinto et al. (2022) found significantly greater entrainment at the word frequency (and its 3^rd^ harmonic frequency) and significantly less entrainment at the syllable frequency, when comparing the experimental and control condition.

Furthermore, attention does not seem to alter the entrainment to the word frequency, although syllable entrainment is greater when attention is directed towards the stimuli (Batterink & Paller, 2019), and both typical readers and dyslexic readers were found to entrain to the word frequency (Zhang et al., 2021), although at different paces. Furthermore, item-level entrainment at the word frequency was higher for word items that were subsequently better recognized (Batterink, 2020).

Perfectly estimating at which point the entrainment reaches a significant effect is challenging as the studies typically compared exposure blocks of varying duration. Generally speaking, the effect seems to develop rather rapidly, typically within the first couple of minutes of exposure to the learning stream.

#### Evidence of the association

Entrainment to the underlying structures was significantly associated with the learning outcome in 6 out of 11 cases. However, some of these results do not directly support the idea of entrainment to the word frequency as a potential predictor of individual learning outcome. Namely, in Kabdebon et al. (2015), only entrainment to the syllable frequency, not the word frequency, was correlated to the learning outcome. Pinto et al. (2022) did not observe a significant correlation when assessing the average individual entrainment at the word frequency and its harmonics to the postlearning tasks. Instead, a significant difference was observed when comparing participants that showed entrainment to the word frequency to participants that did not (although only for reaction time of the target-detection task).

Three of the studies that found a significant association included several postlearning tasks, and all of them found that reaction time on the target-detection task was the only measure significantly associated with entrainment (Batterink & Paller, 2017, 2019; Pinto et al., 2022). For Pinto et al. (2022), this was the case for the group comparison, but not at the individual level. In general, indirect measures are most often predicted by individual entrainment to the word frequency (reaction time on target-detection task for adults, and preferential looking time for infants), and the syllable frequency (entrainment to part and rule words for infants), in this pool of studies. Explicit measures, namely performance on the rating task and 2AFC task, were only predicted by individual participants’ entrainment to the word frequency in Batterink (2020; for 2AFC). As such, the type of postlearning measure might play a role in whether an association between entrainment and learning outcome is found. As highlighted in several of these studies (most prominently in Pinto et al., 2022), low intraindividual correlations between different behavioral measures are commonly observed in SL experiments.

The results of 3 studies are in support of WLI as a predictor of the learning outcome (for implicit measures—namely, RT on the target-detection task and preferential looking-time with infants; Batterink & Paller, 2017, 2019; Choi et al., 2020). Moreau et al. (2022), however, did not find the WLI to predict learning outcome for either children or adults. Typically, the WLI is found to increase over time only in the experimental condition. Interestingly, however, the WLI for the experimental and the control conditions have been found to be highly correlated, and the WLI of the control is equally (or in fact somewhat more strongly) correlated with the learning outcome (Batterink & Paller, 2017). What is actually captured by the WLI is, as such, somewhat unclear.

Significant associations were not found between entrainment and the learning outcome in five studies (or in six cases, if Moreau & colleagues, 2022, are counted twice, as they assessed both adults and children). Overall, all included studies share striking resemblance to each other in terms of experimental design, although, there is some variability in what is being entered into the correlation analysis or prediction model, in terms of the entrainment and postlearning measures. Entrainment measures that have been used include the WLI, maximum entrainment at the word frequency, entrainment at the word and syllable frequencies, entrainment to the word frequency for the last part of learning subtracted by the first part, average entrainment at the word frequency and its harmonics, or comparing groups in which entrainment was or was not observed. The postlearning measures vary both in terms of explicit versus implicit measures, and which aspect of the test that is being associated with the entrainment (such as reaction time or hit rate on the target-detection task). This might be one explanation for the lack of association in around half of the studies.

For infants and children, some indication of overall entrainment to the word frequency was reported. However, the extent to which this is related to the learning outcome is unclear for several reasons. Firstly, a significant association was not found for children (Moreau et al., 2022). Further, increased entrainment to the syllable frequency, not the word frequency, was associated with the learning outcome in one of the studies with infants (Kabdebon et al., 2015), which does not necessarily speak for sensitivity to the word structures. Lastly, a significant correlation was found between a novelty preference for nonwords and the WLI (Choi et al., 2020), which could speak for some association.

### Summary of findings

In the current scoping review, 11 articles were identified and assessed as eligible for inclusion. Some explored the concept of the online segmentation process (the word-identification component), while others used the online segmentation measure as a tool to study SL and factors that might affect it. All studies included one or more postlearning tests of SL to assess the learning outcome and observed entrainment to the underlying statistical pattern during exposure in the learning phase. Furthermore, entrainment is generally documented regardless of study-specific variability in research design and age of participants. In several studies, entrainment was found to be modulated by the duration of the learning phase. About half of the studies (6/11) did report on a significant association between the entrainment to the underlying structure and the learning outcome.

## Discussion

The current review aimed at scoping the existing literature on the topic of neural entrainment in auditory linguistic SL, providing insight into ways in which the paradigm is currently being used, as well as highlight where the focus of future research can be placed. This approach was chosen based on the relatively low number of available studies, to provide an overview of this emerging subfield of SL.

All the included studies report on some form of entrainment to the statistical pattern—namely, the embedded pseudowords—as well as the syllable rate, indicating that the speech streams were processed at the sensory level. In addition, about half of the studies observed a significant association between neural entrainment and the learning outcome. Thus, it is unclear what the observed entrainment truly represents: whether it is a processing mechanism which can be a prerequisite for learning, or, in fact, a sufficient predictor of the learning. This will be discussed in the following sections to address whether and to what extent neural entrainment is a useful measure of statistical word-learning.

### Entrainment as general processing mechanism and a prerequisite for SL

First, we explore the possibility of neural entrainment representing a preliminary processing mechanism. That is, a precondition, for learning to occur. As the results highlight, the presence of entrainment to the underlying structure might not in itself be evidence of learning taking place, but it could represent early detection of regularities in the input signal, which arguably plays a role in subsequent word retention. In other words, the entrainment could reflect the initial identification of regularities, the word-identification component of SL. However, if entrainment is more of a general trait of human processing of linguistic regularities, individual differences in the word-identification component would not necessarily predict individual differences in the learning outcome.^6^

Some evidence pointing in this direction is the fact that the WLI for the structured stream has been found to highly correlate with the WLI of the control condition (Batterink & Paller, 2017). In other words, individuals who tend to segment the input into the frequency of words also tend to segment a stream of syllables, containing no statistical cues to segmentation, at the same frequency. In several of the studies, peaks in entrainment are in fact also observed at the word frequency in the control condition. However, in a reanalysis of previous results, this peak disappeared compared with the original Batterink and Paller (2017) study, speaking for the importance of methodological considerations taken into account when conducting these studies (Batterink & Choi, 2021; Benjamin et al., 2021). Interestingly, however, the WLI for the structured (*r* = .59, *p* = .003), and the random (*r* = .42, *p* = .039) learning streams were significantly correlated with the learning outcome in both the reanalysis and the original paper (Batterink & Choi, 2021; Batterink & Paller, 2017).

A shift in entrainment from smaller constituents to larger segments of the input signal, might represent a shift from bottom-up processing of each syllable to more top-down processing of words, as the neural system picks up on the regularity. This shift could facilitate a general increase in processing speed of the structured stream. As such, the word-identification component detects and allows for more efficient processing of the statistical patterns embedded in the auditory stimuli, which in turn is a prerequisite for the emergence of explicit knowledge (Moser et al., 2021). However, the actual emergence of explicit knowledge might vary between individuals and could be affected by other cognitive factors such as awareness, attention, and comprehension. In other words, factors that are more related to the memory-storage component (Batterink & Paller, 2017).

Attention, for example, did not alter entrainment to the underlying structure or the time course of entrainment, although participants with full attention towards the stimulus had significantly greater entrainment to the syllable frequency (Batterink & Paller, 2019). These participants also had, behaviorally, significantly faster reaction times on the target detection task (which was correlated with the WLI). In other words, tracking of the word structure was not altered by attention, while the behavioral learning outcome was. Similarly, cognitive depletion as induced with transcranial magnetic stimulation (TMS) and a cognitive fatigue task had the same effect on the learning outcome (i.e., enhanced memory for pseudowords presented during the learning phase, but differential effects on entrainment; i.e., significantly greater WLI in the TMS induced, but not in the cognitive fatigue induced group; Smalle et al., 2022). As pointed out by the authors, this is in line with the view that the word-identification component and the memory storage component are dissociable aspects of SL.

Models of SL which incorporate more than two components might also be useful here,^7^ such as the four-component model suggested by Karuza et al. (2014), which includes (1) sensory input encoding, (2) pattern extraction, (3) model building, and (4) retrieval or recognition. Encoding of the input is not sufficient for learning, but rather a prerequisite. However, memory formation is not guaranteed. Additionally, given the above model, entrainment could potentially reflect either the sensory input encoding component *or* the pattern extraction component, which opens a larger room for interpretation of the results, in terms of why certain associations may or may not be observed in the various studies. Variance in either component, when encoding the representations of individual elements *and/or* when identifying the statistical regularities of those representations, could influence the learning outcome (Frost et al., 2015). These two components could potentially influence each other as well, possibly reflected in the neural signal in different ways, which could, in some cases but not necessarily all, influence the learning outcome. Such an interplay might thus be what is captured by the WLI, which could explain why the WLI for the experimental and control conditions are related, and both correlated with the learning outcome.

Following this line of thinking, individuals who more readily pick up on acoustic cues (i.e., sensory input encoding) could also better detect and extract regularities from the input (i.e., pattern extraction). This is potentially compatible with the results of the studies in which an association between the entrainment and the learning outcome was observed. This would speak for entrainment at the word frequency as a prerequisite for SL.

### Entrainment as general processing mechanism and not a prerequisite for SL

Alternatively, individuals who more readily pick up on acoustic cues could also better detect and extract regularities from the input *regardless* of what the entrainment at the word frequency truly represents. Namely, someone who more effectively picks up on acoustic features (in this scenario reflected by the entrainment at the word frequency) could also be better at detecting statistical regularities (in this scenario reflected in the learning outcome). The fact that the two variables are associated in some of the included studies does not necessarily indicate that they represent the same underlying process. Instead, it could speak for the individual learners’ general effectiveness in picking up cues from the environment, as reflected in their ability to detect acoustic features associated with the word frequency (in this scenario reflected as entrainment to that frequency), as well as in their SL ability (in this scenario reflected in the learning outcome).

Acoustic features in the learning stream associated with the word frequency might in fact play a bigger role in these experiments than what is typically ascribed to them. The overarching rationale of these experiments is that entrainment at the frequency of the embedded structures emerges as a consequence of detecting the underlying statistical regularity, and is not directly derived from some other acoustic features in the signal. Pinto et al. (2022) highlight that the type of stimuli typically used during the learning-phase of these experiments—namely, a continuous stream of syllables with or without a statistical regularity—inherently contains acoustic features at the frequency of the word. These cues emerge from subtle and systematic differences in the envelope shape of different syllables (Pinto et al., 2022). Entrainment to this frequency could thus be an auditory response, similar to the peak observed at the syllable frequency. As pointed out in several studies, a mere peak in entrainment at the word frequency does not necessarily speak for learning on its own (Batterink, 2020; Batterink & Paller, 2019; Pinto et al., 2022), although it is interpreted as such in some studies (Table 2). Few studies have employed an acoustically controlled control condition (Pinto et al., 2022).

Speaking for such an account is the fact that entrainment to the word frequency happens in highly similar ways regardless of the awareness of the underlying pattern or the attention brought to it (Batterink & Paller, 2019; e.g., as indicated by a similar response observed for infants, who cannot be made aware of the underlying word structure; Choi et al., 2020; Kabdebon et al., 2015), and during sleep (Batterink & Zhang, 2022; Fló et al., 2022). Furthermore, acoustic features were addressed in a re-analysis of the data from Batterink and Paller (2017) by van der Wulp (2021). Here, the obligatory contour principle, which is a phonological hypothesis stating that specific phonetic features cannot occur consecutively, was used as a potential explanation of the results. Notably, a constraint of place of articulation was found to explain the observed data equally well as condition (structured vs. random).

Entrainment can thus be interpreted as a more general mechanism which picks up on some acoustic feature(s), rather than the statistical regularity among syllables. Still, this mechanism would likely inform on some aspect of the input signal, which can be useful in predicting upcoming events and reducing uncertainty about the environment more generally. These features might not be as prominent as, for example, syllables, leading the entrainment to develop at a somewhat slower pace (although it typically occurs relatively quickly in the experiments described here). This could thus reflect the initial and later processing of acoustic features of the input, rather than statistical learning per se. As such, it is somewhat problematic that several of the studies use “change over time” as their primary measure of online SL. Although a gradual change over time is considered a hallmark of learning more generally, it is still uncertain whether the change observed here truly represents a mechanism related to SL specifically.

### Entrainment as a predictor of SL

Now turning to another interpretation of these results, in which entrainment *is* sufficient for learning—namely, that individual differences in entrainment influence the learning outcome. Although the robustness of this association cannot be directly assessed in the current review, around half of the studies did observe such an association at the individual level. This is particularly supported by Batterink (2020), in which item-level recognition of words was predicted by entrainment to the word frequency. This is a finding in support of the entrainment as reflective of word learning, rather than, for example, individual sensitivity to temporal rhythms. Although, as mentioned, a mere peak at the word frequency does not necessarily represent learning of the distributional cues in the input signal. However, a peak is in fact observed when compared against an acoustic, position-controlled control condition, although unambiguous predictions could not be made about its association to the learning outcome (Pinto et al., 2022).

Notably, mostly implicit reaction time postlearning tasks, or indirect measures, such as the neural response to the word after learning or looking-times, displayed a significant relationship with entrainment. In many cases, however, other postlearning tests were conducted, but were not associated with entrainment measures. Pinto et al. (2022) found that some predictions *possibly* can be made between entrainment and learning outcome at the group, but not the individual, level. A general trend here, as well as in the SL literature more broadly, is the tendency for participants to not show a significant learning effect (at the group level), or, only a few participants showing significant learning effects (at the individual level; Frost et al., 2015; Isbilen et al., 2020; Pinto et al., 2022; Siegelman & Frost, 2015). Furthermore, these postlearning measures often correlate poorly with each other, or correlate in surprising ways across, for example, age groups (e.g., Moreau et al., 2022; Pinto et al., 2022). This raises the question of what learning effects of any given SL task represent, and what it means when entrainment is correlated to some, but not all, measures of SL.

One reason reaction-time-based measures of SL is predicted by entrainment in some of these studies might be related to a potential increase in overall processing speed, following a shift from bottom-up to top-down processing of the input. This might be better captured by implicit SL tasks, as compared with explicit recognition of the words (Batterink et al., 2015; Moser et al., 2021). However, explicit tasks have previously been criticized for methodological flaws in their capacity to capture statistical learning outcomes, in particular with regards to individual differences (e.g., Isbilen et al., 2020). Inability to find that the learning outcome is predicted by individual differences in entrainment to the word structure (if such an association truly exists), is unsurprising, if the test poorly picks up the learning of the distributional pattern to begin with.

If entrainment to the word frequency reflects early detection of the structures embedded in the learning stream, it might be useful to study this specific process, independently of the association to the learning outcome. A limitation is that there is, as of yet, not enough certainty about what neural entrainment to rhythmic stimuli reflects, in general and in language processing specifically, to truly understand its role in SL. Furthermore, as mentioned above, some acoustic confound appears to be present in the segmentation streams typically used in SL studies (Pinto et al., 2022), which complicates our ability to draw conclusions.

### The usefulness of the approach

The overarching review question of the current scoping review is whether and to what extent neural entrainment is a useful online measure of statistical word-learning. As such, the body of evidence on this topic was identified and assessed, and study-specific characteristics related to factors such as study population, design, the results of the entrainment analysis and the evidence of the association to the learning outcome, was identified and synthesized. As discussed, based on the presented papers, the current evidence for entrainment as a marker of the online segmentation process is inconclusive.

Entrainment might represent a general processing mechanism, which could be necessary but not sufficient for SL to occur. This is further supported by the fact that entrainment, to a certain extent, was observed in all included studies, although a correlation with the learning outcome was observed only in some studies, and that it can happen outside the focus of attention. Furthermore, none of the extracted study-specific factors (i.e., age of study population, type of population, sample size, type of neural recording, number of recording electrodes, type of control condition, type of statistical regularity, type, duration and number of segmentation units, duration of the learning phase, and type of postlearning task) appear to affect the results in any clear direction, although implicit measures of learning might be more strongly associated with the mechanism which entrainment taps into. However, entrainment might also represent a general processing mechanism which is not necessary for SL, and rather be a reflection of the processing of acoustic features of the learning stream. Lastly, it could reflect the online detection of statistical regularities. However, future research is needed to disentangle the potential role of entrainment in SL and its relation to the learning outcome.

#### Limitations

The current review is not without its limitations. The number of studies included is relatively low, which limits the possible conclusions that can be drawn. In addition, there might be unavailable studies or results which are not reported, as they are null-findings. This might skew the interpretation of the results presented. In addition, the focus of the current review has been on auditory linguistic SL paradigms, given the status of SL as a crucial mechanism in language acquisition. However, SL has also been argued to aid the process of extracting statistical regularities from the environment in general. In similar studies, where, for example, statistical tone patterns are learned, comparable results have been found, with regard to entrainment (Moser et al., 2021). Furthermore, the focus here was on measures of phase entrainment, specifically, although we recognize that similar frequency-tagging approaches where power is estimated would provide highly comparable results (for methodological considerations, see Benjamin et al., 2021; van Diepen & Mazaheri, 2018).

#### Research gaps and future directions

The last review objective was to identify research gaps in the extant research. Evidently, there is still much to be learned about (1) what entrainment to word frequency truly represents and (2) how it potentially relates to the learning outcome. To address these points, we encourage studies on the contribution of acoustic confounds to the word-level entrainment, as well as a broader review of entrainment in SL (see Limitations). Furthermore, to assess the strength of the association between entrainment and the learning outcome, a meta-analysis of relevant articles is encouraged. It was, however, not the aim of the current study. If implicit measures of SL are in fact more strongly associated with entrainment, theoretical proposals of why this might be are necessary. Lastly, to accurately estimate the predictive value of online neural entrainment to the learning outcome, reports of null-findings are also needed.

## Conclusions

The current scoping review has aimed to address the relationship between neural entrainment to the underlying pattern of an artificial language and the learning outcome, as measured through postlearning tests, to assess whether and to what extent entrainment is a useful online measure of statistical word-learning. A total of 11 articles were included, where all observed some form of entrainment during the learning phase of the experiments, and about half (6) of the studies found an association between entrainment and the learning outcome. The results of the included studies thus show inequivalent results. That is, no clear conclusions on what the entrainment actually represents can be drawn, although we speculate that it might reflect a general auditory processing mechanism, rather than segmentation of the speech stream per se. Based on the presented results, we suggest a comprehensive meta-analysis of SL studies across modalities, in which entrainment and learning outcome is assessed, as well as research further elaborating on the potential acoustic confounds often associated with these studies.

### Supplementary information

ESM 1 (PDF 20 kb)

## Data Availability

Protocol and protocol updates and search history are available (https://osf.io/agvb4).

## Footnotes

1 known as nonadjacent dependencies.

2 For more technical details and examples of, as well as issues with, the analysis, see, Delorme and Makeig, 2004; Morales & Bowers, 2022; van Diepen & Mazaheri, 2018.

3 We found it useful to structure the content of the current review around the theoretical framework presented in Batterink and Paller (2017), which is why it is described in the Introduction, despite not being the earliest article on the subject nor the only proposed theoretical framework (see Discussion).

4 A harmonic is a wave whose frequency is an integral multiple of the frequency of the same reference wave. Here, the word frequency corresponds to the reference wave (i.e., the 2nd harmonic frequency).

5 Counting Moreau et al. (2022) twice as the association was assessed for both adults and children.

6 Note that in the two-component model outlined by Batterink and Paller (2017), it is not claimed that the word-identification component is responsible for learning alone, but that individual differences in the component could explain some of the variation in learning outcomes.

7 Note that Batterink and Paller (2017) does not limit their suggested model to two components. Rather, it is argued that SL is made up of *at least* two components.

## References

[CR1] Adrian, E. D. (1944). Brain rhythms. *Nature, 153*(3882), 360–362.

[CR2] Batterink, L. J. (2020). Syllables in sync form a link: Neural phase-locking reflects word knowledge during language learning. *Journal of Cognitive Neuroscience, 32*(9), 1735–1748. 32427066 10.1162/jocn_a_01581 PMC7395883

[CR3] Batterink, L. J., & Choi, D. (2021). Optimizing steady-state responses to index statistical learning: Response to Benjamin and colleagues. *Cortex, 142*, 379–388. 34321154 10.1016/j.cortex.2021.06.008

[CR4] Batterink, L. J., & Paller, K. A. (2017). Online neural monitoring of statistical learning. *Cortex, 90*, 31–45. 28324696 10.1016/j.cortex.2017.02.004 PMC5438777

[CR5] Batterink, L. J., & Paller, K. A. (2019). Statistical learning of speech regularities can occur outside the focus of attention. *Cortex, 115*, 56–71. 30771622 10.1016/j.cortex.2019.01.013 PMC6513683

[CR6] Batterink, L. J., Reber, P. J., Neville, H. J., & Paller, K. A. (2015). Implicit and explicit contributions to statistical learning. *Journal of Memory and Language, 83*, 62–78. 26034344 10.1016/j.jml.2015.04.004 PMC4448134

[CR7] Batterink, L. J., & Zhang, S. (2022). Simple statistical regularities presented during sleep are detected but not retained. *Neuropsychologia, 164*, 108106. 34864052 10.1016/j.neuropsychologia.2021.108106

[CR8] Benjamin, L., Dehaene-Lambertz, G., & Fló, A. (2021). Remarks on the analysis of steady-state responses: Spurious artifacts introduced by overlapping epochs. *Cortex, 142*, 370–378. 34311971 10.1016/j.cortex.2021.05.023

[CR9] Buiatti, M., Peña, M., & Dehaene-Lambertz, G. (2009). Investigating the neural correlates of continuous speech computation with frequency-tagged neuroelectric responses. *NeuroImage, 44*(2), 509–519. 18929668 10.1016/j.neuroimage.2008.09.015

[CR10] Choi, D., Batterink, L. J., Black, A. K., Paller, K. A., & Werker, J. F. (2020). Preverbal infants discover statistical word patterns at similar rates as adults: Evidence from neural entrainment. *Psychological Science, 31*(9), 1161–1173. 32865487 10.1177/0956797620933237

[CR11] Cunillera, T., Càmara, E., Toro, J. M., Marco-Pallares, J., Sebastián-Galles, N., Ortiz, H., Pujol, J., & Rodríguez-Fornells, A. (2009). Time course and functional neuroanatomy of speech segmentation in adults. *NeuroImage, 48*(3), 541–553. 19580874 10.1016/j.neuroimage.2009.06.069

[CR12] Delorme, A., & Makeig, S. (2004). EEGLAB: An open source toolbox for analysis of single-trial EEG dynamics including independent component analysis. *Journal of Neuroscience Methods, 134*(1), 9–21. 15102499 10.1016/j.jneumeth.2003.10.009

[CR13] Ding, N., Melloni, L., Zhang, H., Tian, X., & Poeppel, D. (2016). Cortical tracking of hierarchical linguistic structures in connected speech. *Nature Neuroscience, 19*(1), 158–164. 26642090 10.1038/nn.4186 PMC4809195

[CR14] Elmer, S., Valizadeh, S. A., Cunillera, T., & Rodriguez-Fornells, A. (2021). Statistical learning and prosodic bootstrapping differentially affect neural synchronization during speech segmentation. *NeuroImage, 235, Article 118051*. 10.1016/j.neuroimage.2021.118051 33848624

[CR15] EndNote. (2013). *EndNote* (Version 21) [64 bit]. Clarivate.

[CR16] Fló, A., Benjamin, L., Palu, M., & Dehaene-Lambertz, G. (2022). Sleeping neonates track transitional probabilities in speech but only retain the first syllable of words. *Scientific Reports, 12*(1), Article 4391. 35292694 10.1038/s41598-022-08411-w PMC8924158

[CR17] Frost, R., Armstrong, B. C., Siegelman, N., & Christiansen, M. H. (2015). Domain generality versus modality specificity: The paradox of statistical learning. *Trends in Cognitive Sciences, 19*(3), 117–125. 25631249 10.1016/j.tics.2014.12.010 PMC4348214

[CR18] Getz, H., Ding, N., Newport, E. L., & Poeppel, D. (2018). Cortical tracking of constituent structure in language acquisition. *Cognition, 181*, 135–140. 30195135 10.1016/j.cognition.2018.08.019 PMC6201233

[CR19] Giraud, A.-L., & Poeppel, D. (2012). Cortical oscillations and speech processing: Emerging computational principles and operations. *Nature Neuroscience, 15*(4), 511–517 10.1038/nn.3063 22426255 PMC4461038

[CR20] Goswami, U. (2019). Speech rhythm and language acquisition: An amplitude modulation phase hierarchy perspective. *Annals of the New York Academy of Sciences, 1453*(1), 67–78. 31237357 10.1111/nyas.14137

[CR21] Henin, S., Turk-Browne, N. B., Friedman, D., Liu, A., Dugan, P., Flinker, A., Doyle, W., Devinsky, O., & Melloni, L. (2021). Learning hierarchical sequence representations across human cortex and hippocampus. *Science*. *Advances, 7*(8), Article eabc4530. 10.1126/sciadv.abc4530 PMC7895424 33608265

[CR22] Hunt, R. H., & Aslin, R. N. (2001). Statistical learning in a serial reaction time task: Access to separable statistical cues by individual learners. *Journal of Experimental Psychology: General, 130*(4), 658. 11757874 10.1037//0096-3445.130.4.658

[CR23] Isbilen, E. S., & Christiansen, M. H. (2022). Statistical learning of language: A meta-analysis into 25 years of research. *Cognitive Science, 46*(9), e13198. 36121309 10.1111/cogs.13198

[CR24] Isbilen, E. S., McCauley, S. M., Kidd, E., & Christiansen, M. H. (2020). Statistically induced chunking recall: A memory-based approach to statistical learning. *Cognitive Science, 44*(7), e12848. 32608077 10.1111/cogs.12848

[CR25] Kabdebon, C., Peña, M., Buiatti, M., & Dehaene-Lambertz, G. (2015). Electrophysiological evidence of statistical learning of long-distance dependencies in 8-month-old preterm and full-term infants. *Brain and Language, 148*, 25–36. 25865749 10.1016/j.bandl.2015.03.005

[CR26] Karuza, E. A., Emberson, L. L., & Aslin, R. N. (2014). Combining fMRI and behavioral measures to examine the process of human learning. *Neurobiology of Learning and Memory, 109*, 193–206. 24076012 10.1016/j.nlm.2013.09.012 PMC3963805

[CR27] Karuza, E. A., Newport, E. L., Aslin, R. N., Starling, S. J., Tivarus, M. E., & Bavelier, D. (2013). The neural correlates of statistical learning in a word segmentation task: An fMRI study. *Brain and Language, 127*(1), 46–54. 23312790 10.1016/j.bandl.2012.11.007 PMC3750089

[CR28] Kidd, E., Arciuli, J., Christiansen, M. H., Isbilen, E. S., Revius, K., & Smithson, M. (2020). Measuring children’s auditory statistical learning via serial recall. *Journal of Experimental Child Psychology, 200*, 104964. 32858420 10.1016/j.jecp.2020.104964

[CR29] Kuhl, P. K. (2004). Early language acquisition: Cracking the speech code. *Nature Reviews Neuroscience, 5*(11), 831–843. 15496861 10.1038/nrn1533

[CR30] Lehiste, I. (1960). An acoustic–phonetic study of internal open juncture. *Phonetica, 5*(s1), 5–54.

[CR31] Lopez-Barroso, D., de Diego-Balaguer, R., Cunillera, T., Camara, E., Muente, T. F., & Rodriguez-Fornells, A. (2011). Language learning under working memory constraints correlates with microstructural differences in the ventral language pathway. *Cerebral Cortex, 21*(12), 2742–2750. 21527790 10.1093/cercor/bhr064

[CR32] Maye, J., Werker, J. F., & Gerken, L. (2002). Infant sensitivity to distributional information can affect phonetic discrimination. *Cognition, 82*(3), B101–B111. 11747867 10.1016/s0010-0277(01)00157-3

[CR33] McNealy, K., Mazziotta, J. C., & Dapretto, M. (2006). Cracking the language code: Neural mechanisms underlying speech parsing. *Journal of Neuroscience, 26*(29), 7629–7639. 16855090 10.1523/JNEUROSCI.5501-05.2006 PMC3713232

[CR34] Morales, S., & Bowers, M. E. (2022). Time-frequency analysis methods and their application in developmental EEG data. *Developmental Cognitive Neuroscience, 54*, 101067. 35065418 10.1016/j.dcn.2022.101067 PMC8784307

[CR35] Moreau, C. N., Joanisse, M. F., Mulgrew, J., & Batterink, L. J. (2022). No statistical learning advantage in children over adults: Evidence from behaviour and neural entrainment. *Developmental Cognitive Neuroscience, 57*, 101154. 36155415 10.1016/j.dcn.2022.101154 PMC9507983

[CR36] Moser, J., Batterink, L., Hegner, Y. L., Schleger, F., Braun, C., Paller, K. A., & Preissl, H. (2021). Dynamics of nonlinguistic statistical learning: From neural entrainment to the emergence of explicit knowledge. *NeuroImage, 240, Article 118378*. 10.1016/j.neuroimage.2021.118378 PMC8456692 34246769

[CR37] Ordin, M., Polyanskaya, L., Soto, D., & Molinaro, N. (2020). Electrophysiology of statistical learning: Exploring the online learning process and offline learning product. *European Journal of Neuroscience, 51*(9), 2008–2022. 31872926 10.1111/ejn.14657

[CR38] Peña, M., Bonatti, L. L., Nespor, M., & Mehler, J. (2002). Signal-driven computations in speech processing. *Science,**298*(5593), 604–607. 12202684 10.1126/science.1072901

[CR39] Pinto, D., Prior, A., & Zion Golumbic, E. (2022). Assessing the sensitivity of EEG-based frequency-tagging as a metric for statistical learning. *Neurobiology of Language, 3*(2), 214–234. 37215560 10.1162/nol_a_00061 PMC10158570

[CR40] Ramos-Escobar, N., Segura, E., Olivé, G., Rodriguez-Fornells, A., & François, C. (2021). Oscillatory activity and EEG phase synchrony of concurrent word segmentation and meaning-mapping in 9-year-old children. *Developmental Cognitive Neuroscience, 51*, 101010. 34461393 10.1016/j.dcn.2021.101010 PMC8403737

[CR41] Saffran, J. R. (2003). Statistical language learning: Mechanisms and constraints. *Current Directions in Psychological Science, 12*(4), 110–114.

[CR42] Saffran, J. R., Aslin, R. N., & Newport, E. L. (1996a). Statistical learning by 8-month-old infants. *Science, 274*(5294), 1926–1928. 8943209 10.1126/science.274.5294.1926

[CR43] Saffran, J. R., & Kirkham, N. Z. (2018). Infant statistical learning. *Annual Review of Psychology, 69*, 181–203. 28793812 10.1146/annurev-psych-122216-011805 PMC5754249

[CR44] Saffran, J. R., Newport, E. L., & Aslin, R. N. (1996b). Word segmentation: The role of distributional cues. *Journal of Memory and Language, 35*(4), 606–621.

[CR45] Saffran, J. R., Newport, E. L., Aslin, R. N., Tunick, R. A., & Barrueco, S. (1997). Incidental language learning: Listening (and learning) out of the corner of your ear. *Psychological Science, 8*(2), 101–105.

[CR46] Schiavo, J. K., & Froemke, R. C. (2019). Capacities and neural mechanisms for auditory statistical learning across species. *Hearing Research, 376*, 97–110. 30797628 10.1016/j.heares.2019.02.002 PMC6456437

[CR47] Shi, R., & Werker, J. F. (2001). Six-month-old infants’ preference for lexical words. *Psychological Science, 12*(1), 70–75. 11294231 10.1111/1467-9280.00312

[CR48] Siegelman, N., & Frost, R. (2015). Statistical learning as an individual ability: Theoretical perspectives and empirical evidence. *Journal of Memory and Language, 81*, 105–120. 25821343 10.1016/j.jml.2015.02.001 PMC4371530

[CR49] Smalle, E. H., Daikoku, T., Szmalec, A., Duyck, W., & Möttönen, R. (2022). Unlocking adults’ implicit statistical learning by cognitive depletion. *Proceedings of the National Academy of Sciences, 119*(2), e2026011119. 10.1073/pnas.2026011119 PMC8764693 34983868

[CR50] Tricco, A. C., Lillie, E., Zarin, W., O’Brien, K. K., Colquhoun, H., Levac, D., Moher, D., Peters, M. D., Horsley, T., & Weeks, L. (2018). PRISMA extension for scoping reviews (PRISMA-ScR): Checklist and explanation. *Annals of Internal Medicine, 169*(7), 467–473. 30178033 10.7326/M18-0850

[CR51] Turk-Browne, N. B., Jungé, J. A., & Scholl, B. J. (2005). The automaticity of visual statistical learning. *Journal of Experimental Psychology: General, 134*(4), 552. 16316291 10.1037/0096-3445.134.4.552

[CR52] van Bree, S., Sohoglu, E., Davis, M. H., & Zoefel, B. (2021). Sustained neural rhythms reveal endogenous oscillations supporting speech perception. *PLOS Biology, 19*(2), e3001142. 33635855 10.1371/journal.pbio.3001142 PMC7946281

[CR53] van der Wulp, I. (2021). *Word segmentation: TP or OCP? A re-analysis of Batterink & Paller (2017)*. https://osf.io/gu7xb/

[CR54] van Diepen, R. M., & Mazaheri, A. (2018). The caveats of observing inter-trial phase-coherence in cognitive neuroscience. *Scientific Reports, 8*(1), Article 2990. 29445210 10.1038/s41598-018-20423-z PMC5813180

[CR55] Zhang, M., Riecke, L., & Bonte, M. (2021). Neurophysiological tracking of speech-structure learning in typical and dyslexic readers. *Neuropsychologia, 158*, 107889. 33991561 10.1016/j.neuropsychologia.2021.107889

