# Supplementary material for: The relationship between neural phase entrainment and statistical word-learning: A scoping review
Source: Psychon Bull Rev. 2023 Dec 7;31(4):1399–419. doi: 10.3758/s13423-023-02425-9 (PMC11358248; doi:10.3758/s13423-023-02425-9)
Supplement: Supplementary file 1 — (PDF 20 kb) [file 13423_2023_2425_MOESM1_ESM.pdf]

Supplementary Materials

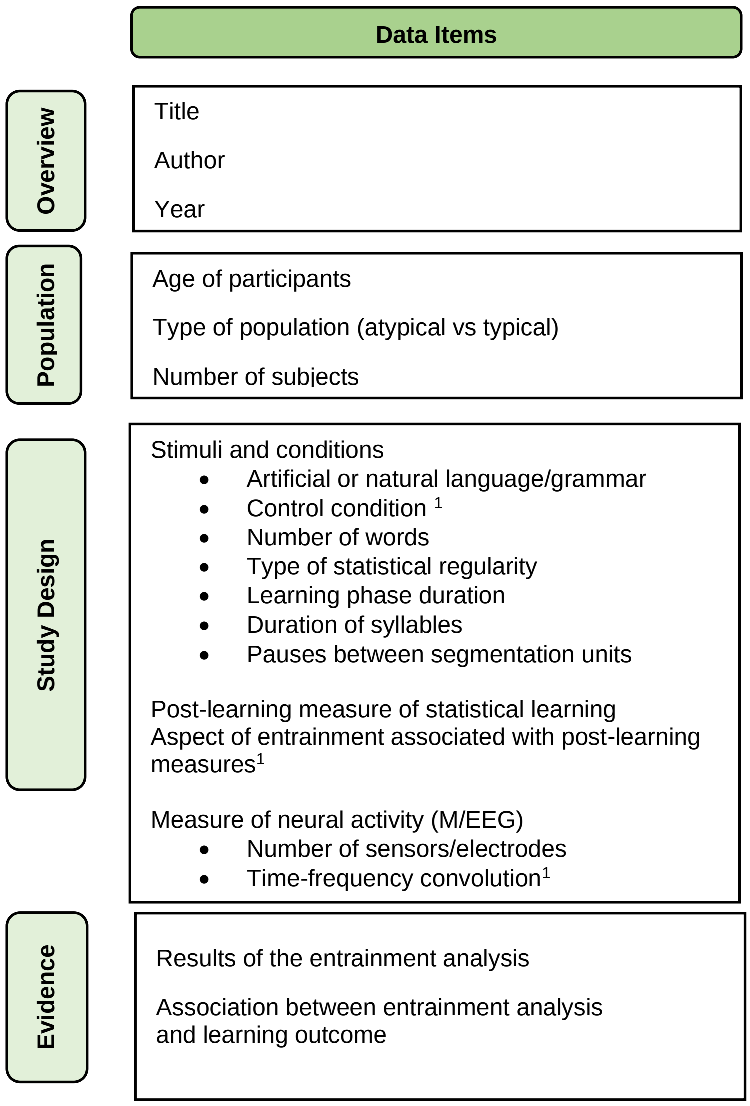

Figure 1. Data extraction template. [1]Note that the following items were added in response to suggestions and comments made by the reviewers during the peer review process.
